# 3-(2-Chloropropyl amide)-4-methoxy-N-phenylbenzamide inhibits expression of HPV oncogenes in human cervical cancer cell

**DOI:** 10.1186/s12985-017-0806-5

**Published:** 2017-07-28

**Authors:** Fang Han, Yanping Li, Qiaoni Lu, Linlin Ma, Huiqiang Wang, Jiandong Jiang, Zhuorong Li, Yuhuan Li

**Affiliations:** 1 Institute of Medicinal Biotechnology, Chinese Academy of Medical Science & Peking Union Medical College, No 1, Tiantan Xili, Beijing, 100050 People’s Republic of China; 2 Institute of Materia Medica, Chinese Academy of Medical Sciences and Peking Union Medical College, No 1, Tiantan Xili, Beijing, 100050 China; 3 Key Laboratory of Molecular Imaging of Shanghai Education Commission, Shanghai University of Medicine & Health Sciences, No 279, Zhouzhugong Road, Shanghai, 201318 People’s Republic of China

**Keywords:** Antiviral, 3-(2-Chloropropyl amide)-4-methoxy-N-phenylbenzamide, Human papillomavirus (HPV) type 16, Cervical carcinoma

## Abstract

**Background:**

Human papillomaviruses (HPVs) are the primary causative agents for cervical cancer, and HPV oncoproteins E6 and E7 are known to be the main reason for the onset and maintenance of the malignancies. Therefore, inhibition of viral E6 and E7 oncoproteins expression represents a viable strategy to cervical cancer therapies. This study is to evaluate the antiviral effect of a novel N-Phenylbenzamide derivative, 3-(2-Chloropropyl amide)-4-methoxy-N-phenylbenzamide (L17), against HPV16 in vitro and identify its associated mechanism of action in cervical cancer cells.

**Methods:**

The cytotoxic effect of L17 was assessed by MTT assay. The mRNA and protein levels of E6 and E7 oncogenes were analyzed by quantitative real-time reverse transcription PCR (qRT-PCR) and Western blot, respectively. p53 and Rb protein levels were also detected by Western blot. The effect of L17 on cell cycle was analyzed by flow cytometry.

**Results:**

The cytotoxic effect of L17 was greater in cervical carcinoma cells than in normal cells. L17 significantly reduced the expression of HPV16 E6 and E7 mRNA and protein, at least partly by enhancing degradation of HPV16 E6 and E7 mRNA. Moreover, reduced expression of E6 and E7 induced by L17 resulted in the up-regulation of p53 and Rb expression, which subsequently induced CaSki cells arrest at G_0_/G_1_ phase.

**Conclusions:**

L17 has antiviral activity through suppressing E6 and E7 oncogene expression and could inhibit CaSki cell proliferating by inducing cells arrest at G_0_/G_1_ phase at nontoxic concentration, implying that L17 might be exploited as a candidate agent for HPV-associated cervical cancer prevention and treatment.

## Background

Cervical cancer is one of the leading causes of cancer death in female [1, 2]. Approximately 99.7% of cervical cancers are caused by high-risk (HR) human papillomavirus (HPV), a small double-stranded DNA virus [3–6]. Although HPV vaccines have been shown to be effective, they only offer prophylactic protection against a minor fraction of HPV serotypes, and have no therapeutic effect for existing HPV infections [7]. In addition, there is no approved antiviral drug for the treatment of HPV infection [8, 9]. The current therapy relies on non-specific removal of infected tissue by often painful ablative procedures [8]. While this strategy may allow for elimination of signs and symptoms, recurrence rates are high due to subclinical virus infection of adjacent tissue [10]. What’s more, for patients with cervical lesions, this strategy may have negative effects on the future reproductive outcomes [11]. These highlight an urgent need for development of efficacious virus-specific inhibitors to overcome HPV-associated cervical cancer.

Two viral oncoproteins E6 and E7 are the main reasons for the development of cervical cancer through binding to two tumor suppressor proteins, p53 and Rb, and neutralizing their functions [12, 13]. E6 protein can facilitate tumor suppressor protein p53 degradation via the ubiquitin proteolytic pathway, which shortens the half-life of p53 and reduces its concentration, leading to the overrides at the G_1_/S and G_2_/M checkpoints [14, 15]. This is the major cause of chromosomal instability and thus leads to the mutation of the HPV-positive cells. Similarly, E7 oncoprotein induces the ubiquitin-mediated proteolysis, and disrupts its association with the E2F family of transcription factors via interacting with the tumor suppressor protein Rb, which subsequently activates genes associated with cell cycle progression [16, 17]. Therefore, inhibition of viral E6 and E7 oncoprotein expression represents a viable strategy that might restore growth control in tumor cells or sensitize cells to cancer therapies.

In our screening for drug candidates exhibiting inhibitory activity against HPV, we found that a novel N-Phenylbenzamide derivative, 3-(2-Chloropropyl amide)-4-methoxy-N-phenylbenzamide, named L17, was active in inhibiting the expression of HPV oncoproteins E6 and E7. This is the first report of the anti-HPV activity of the N-phenylbenzamide derivative, to the best of our knowledge. The main objective of the present study is to evaluate the antiviral effect of L17 against HPV16 in vitro and identify its associated mechanism of action in cervical cancer cells.

## Methods

### Drug

**Fig. 1:**
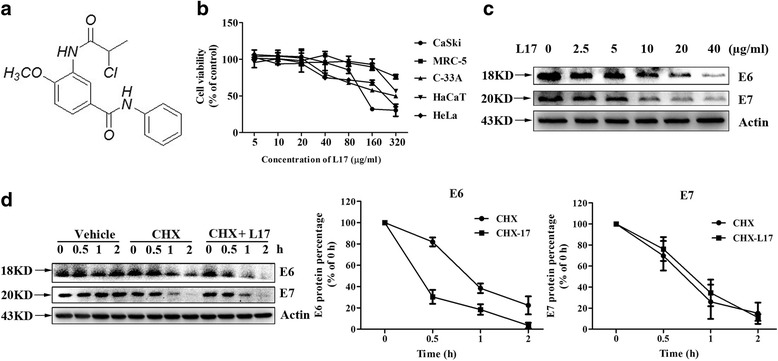
L17 dose-dependently inhibited the expression of E6 and E7 proteins in CaSki cells. **a** The chemical structure of L17. **b** MTT assays for CaSki, HeLa, C-33A, HaCaT and MRC-5 cells. **c** L17 reduced the expression of HPV16 E6 and E7 proteins in CaSki cells by western blot assay. **d** The effect of L17 on the degradation of HPV16 E6 and E7 proteins by western blot assay. Data represent as mean ± SD

L17 (HPLC>98%) was synthesized in Chinese Academy of Medical Sciences and Peking Union Medical College and its chemical structure is shown in Fig. 1a. The compound was dissolved in Dimethyl sulfoxide (DMSO) at 100 mg/ml as a stock solution and further diluted in culture medium prior to use.

### Cell culture

The CaSki (human cervical cancer cells, HPV 16 positive), HeLa (human cervical cancer cells, HPV 18 positive), C-33A (human cervical cancer cells, HPV negative), HaCaT (Human keratinocyte cells, HPV negative) and the MRC-5 (human lung fibroblast cells, HPV negative) were obtained from American Type Culture Collection (ATCC). CaSki cells were cultured in RPMI 1640 media (Invitrogen, Carlsbad, CA, USA), HaCaT cells were cultured in Dulbecco’s Modified Eagle Medium (DMEM, Invitrogen); C-33A, HeLa and MRC-5 cells were cultured in minimum essential medium (MEM, Invitrogen), respectively, containing 10% Fetal Bovine Serum (Gibco, Grand Island, NY, USA), 100 U/ml penicillin G and 100 mg/ml streptomycin [18]. All cells were incubated in humidified atmosphere containing 5% CO_2_ at 37 °C.

### Cytotoxicity assay

The cytotoxic effects of L17 on CaSki, HeLa, C-33A, HaCaT and MRC-5 cells were determined with the 3-(4,5-dimethylthiazole-2-yl)-2,5-biphenyl tetrazolium bromide (MTT, Promega, Madison, WI, USA) [19]. Briefly, CaSki cells (1 × 10^4^ per well) HeLa (2 × 10^4^ per well), C-33A (2 × 10^4^ per well), HaCaT (1.5 × 10^4^ per well) and MRC-5 cells (4.5 × 10^4^ per well) were seeded into 96-well culture plates and incubated for 12 h. Then, different concentrations of L17 were applied in duplicate and incubated for 48 h. 20 μl MTT (5 mg/ml) was added to each well and incubated for another 4 h, followed by solubilization in 150 μl DMSO (Promega) and spectrophotometric measurement at 490 nm on Enspire (Perkin Elmer, Waltham, MA, USA). The maximum non-toxic concentration was defined as the concentration that cell survival rate is greater than 90% (survival rate = absorbance of test group/absorbance of untreated controls).

### Quantitative real-time reverse transcription PCR (qRT-PCR) analysis of HPV16 E6 and E7 transcripts

CaSki cells (6 × 10^5^ per well) were seeded into 6-well culture plates and incubated for 12 h. Then, various concentrations of L17 were applied in duplicate and incubated for 12, 24, 36 and 48 h. Total cellular RNA was extracted with RNeasy Mini kit (Qia-gen, Germantown, MD, USA) according to the manufacturer’s protocol. The one-step quantitative RT-PCR (qRT-PCR) was conducted with the ABI 7500 Fast RT-PCR system (Applied Biosystems, Foster City, CA, USA) using SuperScript III Platinum SYBR Green One-step RT-PCR Kit (Invitrogen, Carlsbad, California, USA) with the following procedures: 50 °C for 3 min, 95 °C for 15 min, followed by 40 cycles of 95 °C for 15 s, 60 °C for 30s [20]. The mRNA expression of HPV16 E6 was determined using primers (F: 5′-CTGCAATGTTTCAGGACCCA-3, R: 5′-TCATGTATAGTTGTGCAGCTCTGT-3′) targeting HPV-16 E6 open-reading frame. The mRNA expression of HPV16 E7 was determined using primers (F: 5′-GAGGAGGAGGATGAAATAGATGGT-3′,R: 5′-CACTTGCAACAAAACGTT ACAATATTG-3′) targeting HPV-16 E7 open-reading frame. β-actin was determined using primers (F: 5′- CCAACCGCGAGAAGATGA-3′, R: 5′- CCAGAGGCGTACAGGGATAG -3′). The ΔΔCt method was used to represent mRNA fold change [21].

### Western blot analysis

CaSki cells (6 × 10^5^ per well) and MRC-5 cells (5 × 10^5^ per well) were seeded into 6-well culture plates and incubated for 12 h. Then, various concentrations of L17 were applied in duplicate and incubated for 48 h. After incubation, whole cell lysates of CaSki and MRC-5 cells were extracted with M-PER mammalian protein extraction reagent (Thermo Fisher Scientific, Waltham, MA, USA). Nuclear fractions of CaSki cells were isolated with NE-PER nuclear and cytoplasmic extraction kit (Beyotime Biotechnology, Shanghai, China). The protein concentrations were determined by the BCA reagents (Thermo Fisher Scientific). The samples, containing 20 μg protein, were boiled for 10 min to denature and resolved on 12% (*w*/*v*) sodium dodecyl sulfate polyacrylamide gel electrophoresis (SDS-PAGE) gels. Then the proteins were transferred to polyvinylidene fluoride (PVDF) membranes (Milli-pore, Billerica, MA, USA). Immunodetection was performed with HPV16-E6 antibody (1:500) (Santa Cruz, sc-460, mouse monoclonal antibody, Dallas, Texas, USA) [22], HPV16-E7 antibody (1:500) (Santa Cruz, sc-6981, mouse monoclonal antibody) [23], p53 antibody (1:1000) (BD Biosciences, San Diego, CA, USA), Rb antibody (1:1000) (BD Biosciences) and Histone H3 antibody (1:1000) (Cell Signaling Technology, Beverly, MA, USA), followed by incubation with HRP-conjugated antibody (1:5000) (Santa Cruz). β-actin (1:5000) (Cell Signaling Technology) was used as a normalization standard in whole cell lysate fractions. Histone H3 was used to normalize nuclear protein loading [20, 24].

### Measurement of stability of HPV16 E6 and E7 mRNAs

RNA synthesis inhibitor actinomycin D (Sigma-Aldrich, St Louis, MO, USA) was used to determine the effect of L17 on the stability of HPV16 E6 and E7 mRNAs [25]. CaSki cells (6 × 10^5^ per well) grown in 6-well plates were treated with or without L17 (40 μg/ml) for 4 h and then 5 μg/ml actinomycin D was added to 6-well plates. Cells were harvested at 0, 2, 4, 6 and 8 h post-actinomycin D treatment. Total cellular RNA was isolated with RNeasy Mini kit and qRT-PCR analyses of HPV16 E6 and E7 transcript levels were performed. HPV16 E6 and E7 transcript levels before actinomycin D treatment were determined as baselines in the experiment. Since mRNA degradation generally obeys first-order process, the slope for the correlation of E6, E7 mRNA level and time is the degradation constant K_d_ (lnC = *ln*C_0_ –K_d_t, where C_0_ is the mRNA concentration at time zero) and the half-life of E6 and E7 mRNA was equal to 0.693/k_d_ according to previously described method [25–28].

### Measurement of HPV16 E6 and E7 protein stability

Translation inhibitor cyclohexamide (CHX, Sigma-Aldrich, USA) was used to determine the effect of L17 on the stability of HPV16 oncoproteins E6 and E7 [25, 29]. CaSki cells (6 × 10^5^ per well) were treated with L17 (40 μg/ml) in the presence or absence of CHX (50 μg/ml). Cells were harvested at 0, 0.5, 1 and 2 h after CHX treatment, and then cell lysates were extracted for Western blot analysis. In all these experiments, HPV16 E6 and E7 protein levels before CHX treatment were determined as baseline.

### Cell cycle analysis by flow cytometry

Flow cytometry analysis was performed to demonstrate the effect of L17 on cell cycle progression. CaSki cells (4 × 10^5^ per well) and MRC-5 cells (4.5 × 10^5^ per well) cells were plated into 6-well culture plates and incubated for 12 h. Then, various concentrations of L17 were applied in duplicate and incubated for 48 h. Then the cells were harvested and fixed in 70% ethanol at 4 °C for 12 h. Before flow cytometry analysis, cells were stained with 1 ml of PI (15 mg/ml) containing RNase (2.5 mg/ml) (Beyotime Biotechnology). DNA content was determined by a Coulter EPICS XL/XL-MCL Flow Cytometry System (Coulter Corp, Brea, California, USA) and the proportion of cells in a particular phase of cell cycle was determined by ModFitLT software.

### Statistical analyses

All results were expressed as mean ± SD of data obtained from at least triplicate experiments using SPSS software. Statistical analysis was performed by using unpaired, two tailed Student’s t-test. All comparisons were made relative to controls and *p* < 0.05 was considered statistically significant.

## Results

### L17 inhibited the expression of E6 and E7 proteins in Caski cells

The chemical structure of L17 is shown in Fig. 1a. We first studied its effects on the viability of CaSki (human cervical cancer cells, HPV 16 positive), HeLa (human cervical cancer cells, HPV 18 positive), C-33A (human cervical cancer cells, HPV negative), HaCaT (Human keratinocyte cells, HPV negative) and the MRC-5 (human lung fibroblast cells, HPV negative). The results showed that the maximum non-toxic concentration of L17 in CaSki, HeLa, C-33A, HaCaT and MRC-5 were 40, 20, 20, 160 and 80 μg/ml, respectively (Fig. 1b).

Next, we studied the effects of L17 on the expression of HPV oncoproteins E6 and E7, which are associated with the development of cervical cancer. Our results showed that L17 down-regulated the expression of HPV E6 and E7 proteins in a dose-dependent manner in CaSki cells (Fig. 1c).

In order to determine how L17 decreased the level of HPV E6 and E7 proteins, we studied the effect of L17 on the stability of E6 and E7 proteins by using cyclohexamide (CHX), a potent translation inhibitor. Decreased stability of E6 protein was observed in presence of L17, whereas stability of E7 protein largely remained unaltered (Fig. 1d).

### L17 enhanced degradation of E6 and E7 mRNAs

**Fig. 2:**
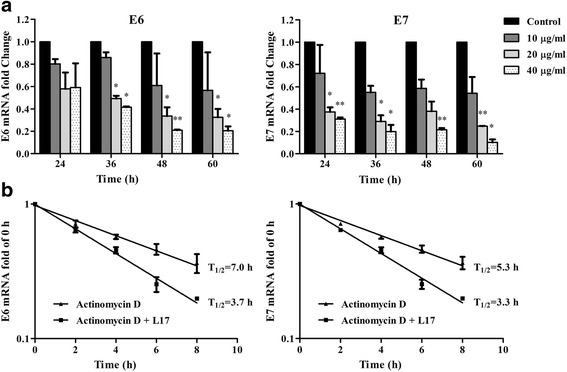
L17 enhanced the degradation of E6 and E7 mRNA. **a** L17 reduced the expression of E6 and E7 mRNA of CaSki cells by one-step qRT-PCR assay. *P < 0.05; **P < 0.01. **b** L17 enhenced the degradation of HPV16 E6 and E7 mRNA by one-step qRT-PCR assay

To examine the effect of L17 on the expression of HPV16 E6 and E7 mRNAs, CaSki cells treated with or without L17 were analyzed by qRT-PCR assay. The results showed that L17 decreased the expression of HPV16 E6 mRNA (Fig. 2a Left) and E7 mRNA (Fig. 2a Right) in a time-dependent and dose-dependent manner.

In order to determine how L17 decreased the HPV16 E6 and E7 mRNA expression, we carried out experiments with actinomycin D, a potent transcription inhibitor. We observed that compound L17 reduced the transcript half-life time from approximately 7.0 h to 3.7 h for E6 (Fig. 2b Left) and from 5.3 h to 3.3 h for E7 (Fig. 2b Right), respectively.

### L17 up-regulated p53 and Rb expression in CaSki cells

**Fig. 3:**
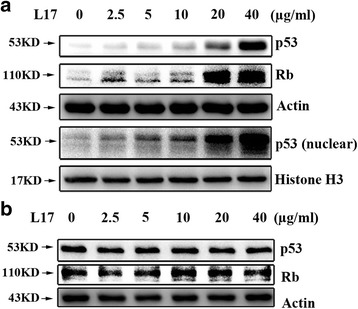
The effect of L17 on the expression of p53 and Rb proteins. **a** L17 up- regulated the expression of total p53 and Rb proteins and p53 in nucleus of CaSki cells by Western blot assay. **b** L17 did not effect the expression of p53 and Rb proteins of MRC-5 cells by Western blot assay

A number of genetic and biochemical studies have shown that p53 and Rb are the most important tumor suppressor proteins in keeping cells from immortalization and transformation [17, 30]. HPV oncoproteins E6 and E7 are known to cause the down-regulation of tumor suppressor proteins p53 and Rb, which is linked to the malignant proliferation of cells [13]. We thus examined the effect of L17 on the expression of p53 and Rb. The results demonstrated that L17 dose-dependently up-regulated p53 and Rb protein expressions in HPV-positive CaSki cells (Fig. 3a). Considering the fact that p53-mediated cell proliferation inhibition depends on its localization in the nucleus [31], it is necessary to examine the effect of L17 on p53 protein level in the nucleus of CaSki cells. We observed that L17 does-dependently increased p53 levels in the nucleus (Fig. 3a), which suggested that p53 exhibited a functional activation in the HPV-positive CaSki cells treated with L17. Interestingly, we found that L17 did not increase p53 and Rb proteins level in HPV-negtive MRC-5 cells (Fig. 3b). These results suggest that reduced expression of E6 and E7 is the key contributor to L17-mediated up-regulation of p53 and Rb.

### The effect of L17 on cell cycle progression

**Fig. 4:**
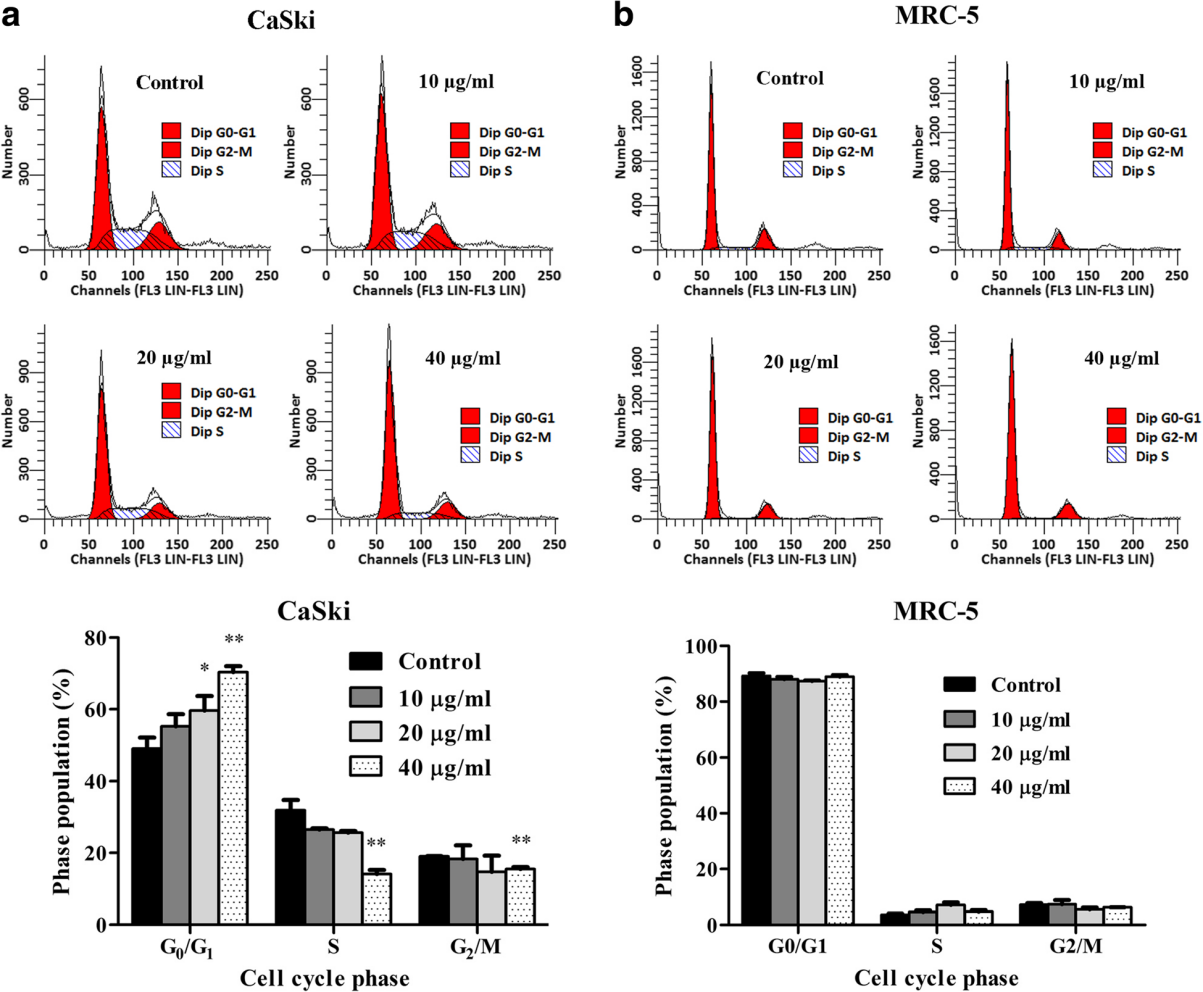
The effect of L17 on cell cycle progression. Flow cytometry analysis of CaSki (**a**) and MRC-5 (**b**) cells. Data represent as mean ± SD. *P < 0.05; **P < 0.01

It has been shown that p53 and Rb played pivotal roles in the negative control of cell cycle progress. Next, we thus performed flow cytometry analysis of PI-stained cells to demonstrate the effect of L17 on cell cycle progression. In line with the increase of p53 and Rb expression in CaSki cells, our results revealed that L17 induced CaSki cells arrest at G_0_/G_1_ phase in a dose-dependent manner (Fig. 4a). Meanwhile, we didn’t observe obvious change of cell cycle in MRC-5 (Fig. 4b) treated with L17, which is in agreement with the finding that L17 had no effect on the levels of p53 and Rb in normal cells.

## Discussion

In our previous study, we have shown that compounds of phenyl benzamide exerted a potent inhibitory action against influenza, EV71 and HCV [32–34]. Herein, we showed for the first time that L17, a novel derivative of phenyl benzamide, exerted a potent inhibitory action against HPV in CaSki cells. Given that persistent infection with high-risk HPVs is the major contributor to cervical cancer. Therefore, this study suggests that L17 might be a potential drug candidate or adjuvant to treat cervical cancer.

It is well established that the HPV oncoproteins E6 and E7 are the main contributors to cervical cancer. Our study demonstrated that L17 dose-dependently inhibited the expression of HPV E6 and E7 proteins in CaSki cells, which confirmed that L17 might have potential inhibitory activity against cervical cancer associated with HPV. Further research found that L17 could significantly decrease the stability of HPV16 E6, E7 mRNA and E6 protein, whereas the stability of E7 protein largely remained unaltered. The mechanisms underlying differential stability of E6 and E7 mRNA are still not understood in this study. It is known that the stability of mRNA is related to miRNA-mediated mRNA degradation, RNA binding proteins and the cell biological state [35–38]. Thus, we speculated that L17-mediated degradation of HPV mRNA might be associated with the changed expression of particular miRNAs targeting E6 and E7 mRNA. However, the exact reason for the decreased stability of E6 and E7 mRNA and protein needs further study.

It is reported that abrogating E6 and E7 function in neoplastic cells by targeting gene expression or protein-protein interaction could reactivate p53 and pRb expression, with subsequent restore cell growth control which includes cell cycle arrest and apoptosis [14–17]. In our study, we observed that L17 does-dependently increased the expression of p53 and Rb tumor suppressor proteins in the HPV16 positive cervical cancer cell line CaSki. At the same time, the tumor suppressor protein levels in the HPV negative cell lines MRC-5 largely remained unaltered. This suggests that L17 does not directly change p53 and Rb expression, and the up-regulation in CaSki is caused by the down-regulation of E6 and E7. The increased nuclear location of p53 in CaSki treated with L17 suggested that p53 had a functional activation. In addition, we found that L17 does-dependently induced G_0_/G_1_ phase arrest in CaSki cells, while had no effect on the cell cycle of MRC-5, which implies that the cell cycle arrest in CaSki by L17 is linked to p53 and Rb up-regulation. In spite of the restoration of p53 function, we did not observe obvious apoptosis in the experiment, which is at odds with other published observations [8, 31]. This probably links to the hypothesis that although L17 could repress the expression of E7 protein, the level of E7 protein in cells probably remains high enough to drive mitotic signals to block the p53-dependent apoptosis.

Human papillomaviruses (HPVs) are the primary causative agents for cervical cancer, and HPV oncoproteins E6 and E7 are known to be the main reason for the onset and maintenance of the malignancies. Our study suggested that L17 had potential action in preventing cervical cancer by reducing the expression of HPV16 E6 and E7 at both the mRNA and protein levels. Importantly, treatment with L17 rescued high levels of p53 and Rb tumoursuppressor proteins in HPV16-positive cervical cells, and then induced the cell cycle arrest at G_0_/G_1_phase. Of note, L17 had no effect on the expression of p53 and Rb and cell cycle in HPV-negative MRC-5. This suggested that L17 did not interfere with the proliferation of normal cells. Compared to direct anticancer drugs, L17 thus reveals a potential advantage of hypotoxicity against normal cell. However, it should be also noted that the lack of apoptosis induction might compromise the ability of L17 inhibition of growth in HPV positive cells.

## Conclusion

In this study, we found that a compound L17 has antiviral activity through suppressing E6 and E7 oncogene expression and might be exploited as a candidate agent for HPV-associated cervical cancer prevention and treatment.

## Abbreviations

CHX: Cyclohexamide
HPVs: Human papillomaviruses
HR: High-risk
L17: 3-(2-Chloropropylamide)-4-methoxy-N- phenylbenzamide
MTT: 3-(4,5-dimethylthiazole-2-yl)-2,5-biphenyl tetrazolium bromide
PVDF: Polyvinylidene fluoride
qRT-PCR: quantitative real-time reverse transcription PCR
SDS-PAGE: Sodium dodecyl sulfate polyacrylamide gel electrophoresis
